# Brain arousal regulation as response predictor for antidepressant therapy in major depression

**DOI:** 10.1038/srep45187

**Published:** 2017-03-27

**Authors:** Frank M. Schmidt, Christian Sander, Marie-Elisa Dietz, Claudia Nowak, Thomas Schröder, Roland Mergl, Peter Schönknecht, Hubertus Himmerich, Ulrich Hegerl

**Affiliations:** 1 Department of Psychiatry and Psychotherapy, University Hospital Leipzig, Semmelweisstr. 10, D-04103, Leipzig, Germany; 2 Research Center of the German Depression Foundation, Leipzig, Germany; 3 Saxonian Hospital Arnsdorf, Arnsdorf, Germany; 4 Institute of Psychiatry, Psychology and Neuroscience, King's College London, London, UK

## Abstract

A tonically high level of brain arousal and its hyperstable regulation is supposed to be a pathogenic factor in major depression. Preclinical studies indicate that most antidepressants may counteract this dysregulation. Therefore, it was hypothesized that responders to antidepressants show a) a high level of EEG-vigilance (an indicator of brain arousal) and b) a more stable EEG-vigilance regulation than non-responders. In 65 unmedicated depressed patients 15-min resting-state EEGs were recorded off medication (baseline). In 57 patients an additional EEG was recorded 14 ± 1 days following onset of antidepressant treatment (T1). Response was defined as a ≥50% HAMD-17-improvement after 28 ± 1 days of treatment (T2), resulting in 29 responders and 36 non-responders. Brain arousal was assessed using the Vigilance Algorithm Leipzig (VIGALL 2.1). At baseline responders and non-responders differed in distribution of overall EEG-vigilance stages (F_2,133_ = 4.780, p = 0.009), with responders showing significantly more high vigilance stage A and less low vigilance stage B. The 15-minutes Time-course of EEG-vigilance did not differ significantly between groups. Exploratory analyses revealed that responders showed a stronger decline in EEG-vigilance levels from baseline to T1 than non-responders (F_2,130_ = 4.978, p = 0.005). Higher brain arousal level in responders to antidepressants supports the concept that dysregulation of brain arousal is a possible predictor of treatment response in affective disorders.

Major depression (MD) is a severe, life threatening and highly prevalent disease^1^ with mostly a recurrent or chronic course. Worldwide, it is one of the leading causes for the medical and economic disease related burden^2^,^3^. Efficient antidepressant psycho- and pharmacotherapies are available – with antidepressants being by far the most often offered treatment option. However, despite antidepressant treatment according to national and international guidelines^4^,^5^, 40–60% of patients show no or only partial response to antidepressant treatment^6^,^7^,^8^. Response predictors to antidepressant therapy in general, to a certain class of antidepressant drugs or for a subgroup of depressed patients would help to avoid current trial-and-error approaches and would be a step towards personalized treatment as well as to a better understanding of the pathomechanisms of MD^9^,^10^.

Different electroencephalographic (EEG) measures have been introduced as potential biomarkers for antidepressant treatment response, such as frequency band power, alpha hemispheric asymmetry, antidepressant treatment response (ATR) index, theta cordance and event-related potentials^11^,^12^,^13^,^14^,^15^,^16^. More recently, the assessment of arousal regulation has become a scope of research on antidepressant treatment response^17^,^18^,^19^ and has been defined as a basic and trans-diagnostically relevant neurobiological dimension within the Research Domain Criteria project (RDoC)^20^. Brain (i.e. central nervous system (CNS)) arousal regulation can best be assessed using EEG-approaches, since different levels of brain arousal are reflected in specific temporo-spatial EEG-patterns. Such arousal levels cannot only be differentiated during sleep (then labelled as sleep stages) but also during wakefulness (then called EEG-vigilance stages). Already small differences or changes in the level of brain arousal will have profound effects on the temporo-spatial pattern of EEG activity upon which most of the previous measures are based^11^,^12^,^13^,^14^,^15^,^16^. Recently the pathogenic relevance of brain arousal regulation for affective disorders has been highlighted^21^. The time-course of EEG-vigilance stages during resting-EEG recordings is a valid indicator of brain arousal regulation. An EEG-based algorithm, the Vigilance Algorithm Leipzig (VIGALL), validated with simultaneous PET-22 and fMRI-analyses^23^, can be used for the objective classification of EEG-vigilances stages. Whereas healthy subjects usually show declines of their arousal level during a 15 minutes resting-EEG with appearance of drowsiness pattern or sometimes even signs of sleep onset, it is a replicated finding that patients with MD in comparison show a tonically high level and more stable regulation of arousal over the whole recording period^24^,^25^. Within the arousal regulation model of affective disorders^21^, a variety of clinical and preclinical arguments have been presented indicating that the upregulation of arousal is a central pathogenic factor in MD. The model also explains several clinical phenomena typically seen in MD such as prolonged sleep onset latencies, avoidance of arousal increasing external stimulation (withdrawal, sensation avoidance) and the response to therapeutic sleep deprivation^26^. Several drugs decreasing wakefulness promoting brain mechanisms (e.g. anticholinergic and antiglutamatergic drugs such as ketamine) have been discussed as possible antidepressants^27^,^28^,^29^,^30^. Additionally, in preclinical studies nearly all established antidepressants have been found to decrease the firing rates of neurons in the locus coeruleus (LC) which might counteract the tonically high brain arousal found in many MD patients. This reduction of LC firing rate was found for acute and for two-week applications of different serotonin-, serotonin-norepinephrine, norepinephrine-, norepinephrine-dopamine reuptake inhibitors, tricyclic antidepressants and monoamine oxidase inhibitors^31^,^32^,^33^,^34^.

Aim of this study is therefore to test the hypotheses that responders to antidepressants compared to non-responders a) show a more tonically high brain arousal level and b) a more stable regulation of brain arousal during 15 minutes of quiet rest as assessed with VIGALL 2.1. Within an exploratory analysis, we further assessed the relationship between early changes of arousal regulation (within 14 ± 1 days following onset of antidepressant treatment) and improvement in depressive symptoms during 28 ± 1 days of antidepressant treatment. Within descriptive analyses, brain arousal of both responders and non-responders were compared to that in healthy controls.

## Results

### Characteristics of groups

At baseline (BL), responders (≥50% improvement in Hamilton Depression Rating Scale (HAMD)-17 after 28 ± 1 days antidepressant treatment) and non-responders were comparable concerning socio-demographic and several clinical aspects (Table 1). Compared to the healthy controls the total group of depressed patients did not differ in age and sex but showed significantly higher scores in Beck Depression Inventory II (BDI-II)-ratings.

### Arousal regulation between responders and non-responders

**Figure 1:**
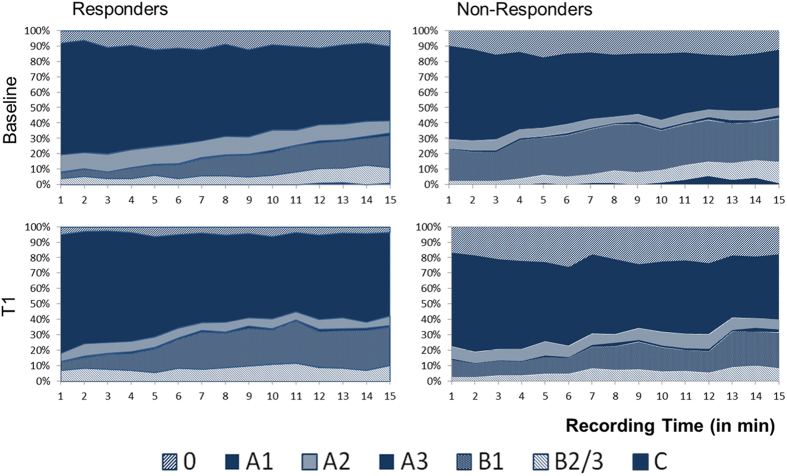
Time-course of EEG-vigilance stages during 15 minutes of resting EEG in responders (N = 29) and non-responders (N = 36) to 4-week antidepressant treatment at baseline and 2 week following onset of treatment (T1).

At BL, significant differences concerning overall occurrence of EEG-vigilance stages were found between responders and non-responders (‘outcome * stage’ interaction: F_2,133_ = 4.780, p = 0.009). Responders spent less time in lower EEG-vigilance stages, as analyses for separate stages (0, A, B, C) as well as sub-stages (A1, A2, A3, B1, B2/3) revealed a more frequent overall occurrence of stages A (Cohen’s d = 0.74) and A1 (Cohen’s d = 0.54) as well as reduced occurrence of lower stages B (Cohen’s d = 0.69) and sub-stage B1 (Cohen’s d = 0.81) within the responders (Table 2, Fig. 1). No differences between response groups were found regarding the time-course of EEG-vigilance stages during the BL-recording period (‘outcome * stage * time’ interaction: F_12,763_ = 0.804, p = 0.648).

ROC analyses examining occurrence of sub-stage B1 as a predictor of response found 0.69 area under the curve (AUC) (*p* = 0.009; 95% CI 0.561–0.819). A B1 cut-off of 15.5% yielded a sensitivity of 69%, a specificity of 69%, a positive predictive value (PPV) of 65% and a negative predictive value (NPV) of 74% to predict response.

**Figure 2:**
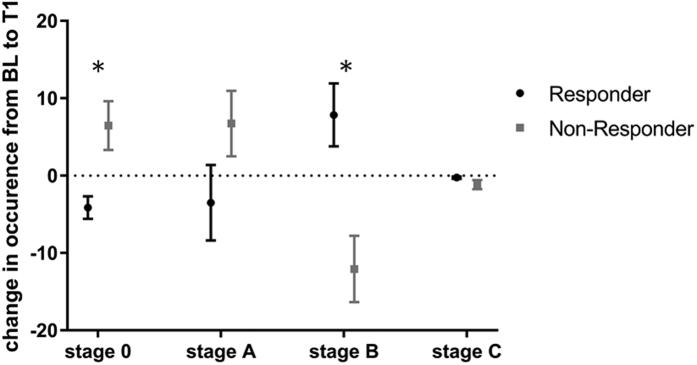
Changes in vigilance stages in percent from baseline (BL) to 2 week following onset of treatment (T1). Responders (N = 27) show decreased occurrence in high vigilance stages 0 and A as well as increases in low vigilance stage B. Non-Responders (N = 30) show increases in stage 0 and A and decreases in stage B. Differences were statistically significant for stage 0, stage B and sub-stage A2 (not shown) and indicated with an asterisk. Error bars are standard errors of the mean.

Investigating changes of EEG-vigilance from BL to T1 revealed a significant decline in overall EEG-vigilance stages in the responders compared to non-responders (‘outcome * stage * assessment’ interaction: F_2,130_ = 4.978, p = 0.005) but no changes in occurrence of EEG-vigilance stages within the total sample (‘stage * assessment’ interaction: F_1,55_ = 3.360, p = 0.072). Analyses on the separate (sub-) stages showed responders to have significantly stronger decreases in sub-stage 0 (Cohen’s d = 0.79) and sub-stage A2 (Cohen’s d = 0.55) as well as increases in stage B (Cohen’s d = 0.89) and sub- stage B1 (Cohen’s d = 0.93) compared to non-responders (Table 3, Fig. 2). ROC analyses examining changes in sub-stage B1 as a predictor of response found 0.79 area under the curve (AUC) (*p* < 0.001; 95% CI 0.673–0.914). A cut-off of 0.23 in change in sub-stage B1 from BL to T1 yielded a sensitivity of 77%, a specificity of 74%, a PPV of 74% and a NPV of 76% to predict response.

Correlation analyses between the changes in the separate (sub-)stages from BL to T1 and changes in HAMD-scores from BL to T2 revealed a significant relationship between reductions in severity of depression and reductions in occurrence of stage 0 (r = 0.380, p = 0.004) as well as an inverse relationship with changes in stage B (r = −0.326, p = 0.013) and sub-stage B1 (r = −0.347, p = 0.008).

### Arousal regulation between depressed patients and healthy controls

Comparing depressed patients to healthy controls, significant differences were found concerning overall occurrence of EEG-vigilance stages (‘group * stage’ interaction: F_2,295_ = 5.461, p = 0.003) but not concerning the time-course of EEG-vigilance stages (‘group * stage * time’ interaction: F_14,1777_ = 1.492, p = 0.106). Post-hoc analyses revealed significantly higher occurrence of stage A (F_1,128_ = 4.490, p = 0.036; Cohen’s d = 0.78), including sub-stage A1 (F_1,128_ = 5.882, p = 0.017; Cohen’s d = 0.42), and lower occurrence of stage B (F_1,128_ = 9.321, p = 0.003; Cohen’s d = 0.53), including sub-stage B1 (F_1,128_ = 9,928, p = 0.002; Cohen’s d = 0.55) in the depressed patients.

Comparing responders and healthy controls, a significant difference in overall occurrence of EEG-vigilance stages (‘group * stage’ interaction: F_2,225_ = 9.319, p < 0.001) was found. Post-hoc analyses revealed a significantly more frequent overall occurrence of stage A (F_1,92_ = 12.375, p < 0.001; Cohen’s d = 0.82) and sub-stage A1 (F_1,92_ = 11.522, p = 0.001; Cohen’s d = 0.77), and lower occurrence of stage B (F_1,92_ = 14.243, p < 0.001; Cohen’s d = 0.90), including sub-stage B1 (F_1,92_ = 15.745, p < 0.001; Cohen’s d = 0.99) in the responders. No differences in time-course of EEG-vigilance were shown (‘group * stage * time’ interaction: F_13,1164_ = 0.978, p = 0.470).

For non-responders versus healthy controls, no differences in overall occurrence of stages (‘group * stage’ interaction: F_2,231_ = 1.181, p = 0.313) or time-course of EEG-vigilance (’group * stage * time’ interaction: F_14,1352_ = 1.321, p = 0.189) were found.

### Analyses in escitalopram monotherapy group

In order to investigate the relationship in EEG-vigilance differences between responders and non-responders in a sample receiving the same antidepressant during the observational period, a sub-sample of responders (n = 18) and non-responders (n = 22) to escitalopram monotherapy were compared. MANOVA analyses for BL revealed significant differences in occurrence of stages between groups (‘group * stage’ interaction F_2,93_ = 8.394, p < 0.001), with significantly higher occurrence of high EEG-vigilance stage A (Cohen’s d = 1.27), including sub-stage A1 (Cohen’s d = 1.07) as well as lower occurrence of lower EEG-vigilance stage 0 (Cohen’s d = 0.67), B (Cohen’s d = 1.03) and sub-stage B1 (Cohen’s d = 1.16) in the responders. No differences in time-course of EEG-vigilance stages between groups were found (group * stage * time’ interaction: F_10,365_ = 0.706, p = 0.713). ROC analyses examining occurrence of sub-stage B1 as a predictor of response found 0.80 area under the curve (AUC) (*p* = 0.001; 95% CI 0.660–0.933). A B1 cut-off of 22.57% yielded a sensitivity of 68%, a specificity of 78%, a PPV of 67% and a NPV of 79% to predict response.

The investigation of changes of EEG-vigilance from BL to T1 revealed a significant decline in overall EEG-vigilance stages in the responders (n = 17) compared to non-responders (n = 20) (‘outcome * stage * assessment’ interaction: F_3,119_ = 8.367, p < 0.001). Analyses on the separate sub-stages showed responders to have significantly higher decreases in stage 0 (F_1,35_ = 4.601, p = 0.039), stage A (F_1,35_ = 8.050, p = 0.008) and A1 (F_1,35_ = 5.322, p = 0.027) as well as increases in stage B (F_1,35_ = 19.886, p < 0.001) and sub-stage B1 (F_1,35_ = 20.895, p < 0.001) compared to non-responders. Correlation analyses between the changes in HAMD-scores from BL to T2 and changes in the separate (sub-)stages from BL to T1 revealed a significant relationship between reductions in severity of depression and reductions in occurrence of stage A (r = 0.411, p = 0.011) and sub-stage A1 (r = 0.423, p = 0.009) as well as an inverse relationship with changes in stage B (r = −0.476, p = 0.003) and sub-stage B1 (r = −0.509, p = 0.001).

## Discussion

EEG-vigilance analyses could confirm the hypothesis that responders to antidepressants show a higher brain arousal level compared to non-responders. During the 15 min EEG recording at baseline, the VIGALL algorithm revealed a more frequent occurrence of the high EEG-vigilance stage A (including sub-stage A1) as well as less low vigilance stages B (including sub-stage B1) in responders compared to non-responders.

Previous analyses on frequency power in relationship to clinical response are worth consideration regarding the current investigation. Corresponding to our results on A-stages, which are defined by dominant alpha band activity, EEG-measures on frequency band activity consistently describe responders to have higher alpha band power at baseline^35^,^36^,^37^. Though not interpreted within the framework of arousal regulation by the authors, these findings portend a relationship between the proportion of high vigilance stages and clinical response to antidepressant treatment. Responders and non-responders did not differ concerning sub-stage B2/3 which is characterized by dominant theta band activity. In line with that, other research groups found no differences in absolute or relative theta power^38^ or a decreased theta power in responders to antidepressants^39^,^40^. Owing to the fact that another study found decreased theta activity to be associated with non-response^41^, the heterogeneity in results was recently explained with the origins of the measured theta activities^14^: the reduced widespread frontal activity within the responders^38^,^39^ was ‘considered most likely’ as a sign of reduced drowsiness which may not be the case in the study investigating frontal midline theta activity^41^. For theta activity specifically assessed within the anterior cingulate cortex, results are again contradictory with both decreased and increased activity to be favourable for treatment outcome^11^,^12^,^13^.

The second hypothesis of a more stable regulation of brain arousal over the 15 minutes recording period for responders compared to non-responders could not be confirmed. Such differences in EEG-vigilance regulation were observed in previous studies comparing patients with MD, mania and healthy subjects^24^,^25^,^42^. This feature may be present amongst different entities of affective disorders but presumably may not be of enough penetrance to differ between sub-groups (responders vs. non-responders) within a sample of subjects suffering from the same disorder. A recent study has investigated changes in vigilance in the ‘International Study to Predict Optimized Treatment Response in Depression’ (iSPOT-D) dataset. For the 15 minutes recording period in an exploratory dataset, responders compared to non-responders showed a steeper decline of CNS-arousal. No significant differences arouse neither for the brain arousal level nor when analyzing the first two minutes which was the length for the iSPOT dataset. Missing differences are potentially driven by an underpowered sample containing only 8 non-responders and 17 responders. Further, comparisons to our findings are difficult to make, given the heterogeneity in the samples, with the definition of response after a short treatment interval of only 2 weeks and a lower HAMD-cutoff than the present. In the iSPOT-D dataset investigating the regulation of arousal but not the arousal level within a short two minutes recording period, responders to a selective serotonin re-uptake inhibitor (SSRI), but not to a serotonin-norepinephrine-reuptake-inhibitor (SNRI)^17^, showed a steeper decline of CNS-arousal than non-responders within the two minutes recording period. When interpreting the findings, it is important to consider that the analyses were performed with the VIGALL 2.0 and since then, the VIGALL 2.1 has been developed and validated (Supplement 1; for more details see http://www.uni-leipzig.de/~vigall/). One reason for the development was to optimize a slight over-classification of sub-stage B2/3 occurring especially in the beginning of the recordings due to non-cephalic electric activity. Another was the non-physiological under-classification of sub-stage A1 in favor of sub-stages A2 and A3. Since the iSPOT analyses were limited to these vulnerable first two minutes performed with the VIGALL 2.0, the results should be considered with caution. Also, it remains unclear if a recording period of two minutes is long enough to draw final conclusions on arousal parameters, as our exploratory analyses of a median vigilance index within three 1-min blocks to compare with the iSPOT-findings could not reveal any relation between response and the arousal regulation or level. Or if decisive information may be lost due to too short recording periods, as other studies^24^,^25^ showed differences between groups to be enhanced in the later course of the recordings.

Our exploratory analysis on changes of EEG-vigilance from BL to T1 showed that responders had stronger declines in vigilance levels during treatment (significant decreases in the high vigilance levels stage 0 and sub-stage A2, significant increases in low vigilance levels stage B and sub-stage B1) compared to non-responders. We could further show that improvements in depression severity were related to reductions in occurrence of the high vigilance stage 0 and increases in the lower stage B, further supporting the hypothesized link between reduction of arousal regulation and improvement in depressive symptomatology. In non-pharmacological antidepressant therapies, current investigations of our research group on EEG-vigilance parameters before and after sleep deprivation (SD) could further observe that the vigilance regulation became more unstable during therapy and that responders to partial SD increasingly reached lower vigilance stages during the course of the resting EEG. One mode of action for the relationship between the decline in arousal during rest and clinical response may be that antidepressants decrease the firing rates of neurons of the LC and the dorsal raphe nucleus^31^,^34^, regions crucial for regulation of wakefulness. At the same time, the clinical efficacy of antidepressants was shown to be partly mediated through the reduction of LC activity^32^,^33^. Therefore, a more pronounced decline of arousal throughout treatment in responders might display a susceptibility to medication in a sub-set of depressed patients that is mediated via the antidepressants’ influence on LC neurons.

In line with previous reports^24^,^25^, the total group of MD patients showed a higher brain arousal level when compared with the healthy controls. Splitting groups according to the therapeutic response, differences were found in the subgroup of responders to AD only. Extending the previous cross-sectional investigations^24^,^25^, this raises the question whether or not upregulated arousal regulation separates a core group of depressed patients responding to AD from atypical depression with hypersomnia or fatigue. The latter often show signs of a downregulated arousal regulation and might respond to psychostimulants^43^,^44^. Concerning the prediction of response to specific antidepressants, the iSPOT-D data set indicated that the group of treatment responders receiving a SSRI showed a faster decline of CNS-arousal than non-responders, whereas those patients effectively treated with the SNRI had an increase in heart rate activity^17^. The ATR index could also be applied both in treatment with a SSRI and a norepinephrine–dopamine reuptake inhibitor (NDRI), given that different ATR thresholds were useful for predicting the response to either escitalopram or bupropion treatment^45^.

Limitations of the current work include a possible selection bias leading to a clinically non-representative cohort, given that other DSM-IV and -V Axis 1 disorders that were excluded have a high co-morbidity with depression. In addition the sample size was too low to allow subgroup analyses for patients with the same antidepressant or those with comorbidities such as anxiety disorders or atypical depressive symptomatology. Finally, the sensitivity and specificity of sub-stage B1 as a predictor to treatment response were moderate and need cross-validation in an independent sample.

In conclusion, this first prospective study on EEG-based vigilance regulation as predictor of treatment outcome could confirm the hypothesis of a higher brain arousal level in responders compared to non-responders to antidepressant treatment. Furthermore, the decline in arousal during treatment was related to an improvement in depression severity. Several of the parameters, especially the proportion of sub-stage B1 at baseline and changes in B1 during the early course of treatment, showed moderate effect sizes and positive and negative predictive value. These findings provide evidence that the assessment of EEG-vigilance before treatment could give information about the likelihood of a certain patient to respond to antidepressants. This information can be integrated in the pro´s and con´s when discussing treatment options with the patient.

## Methods and Materials

### Subjects

The total sample of patients consisted of depressed in- and outpatients consecutively recruited between 02/2012 and 01/2015 from the Department of Psychiatry and Psychotherapy of the University Hospital Leipzig. Inclusion criteria were: age ≥18 years; a diagnosis of MD with a current depressive episode with a baseline score ≥10 in the HAMD-17^46^. Exclusion criteria were: use of centrally active medications (including antidepressants) in the previous 14 days; serious suicide risk; organic mental disorders; illegal drugs and/or alcohol abuse within the past 6 months; schizophrenia, schizotypal and delusional disorders; a history of head injury with loss of consciousness exceeding 1 hour; seizure disorder; acute or chronic infection and major somatic disorders. Extending clinical recordings, investigations of inclusion and exclusion criteria were supported with the Structured Clinical Interview for DSM-IV^47^ (SCID-I). Written informed consent was obtained from all patients. The study was performed according to the Helsinki Declaration and approved by Leipzig University Ethics Committee (#278-11-22082011).

89 depressed patients provided informed consent for participation in the study. Of these patients, 24 had to be excluded from final analyses, resulting in data sets of 65 patients eligible for statistical analyses. Reasons for exclusion of datasets were: 1) patients’ withdrawal from participation or non-attendance to the final assessment (N = 12); 2) evidence for excluding somatic or neurological diseases (N = 3); 3) later diagnosis of DSM-IV Axis 1 disorders other than unipolar depression (N = 3); 4) treatment without antidepressant medication (N = 2); 5); pathological EEG (N = 2); artefacts within more than 15% of the recorded EEG-segments or non-operationality of the VIGALL (N = 2). In addition to the patients, data of 65 sex- and age-matched non-depressed controls were selected from a database consisting of EEG-recordings from community volunteers recruited via announcements in the local newspapers, University’s intranet and internet^48^. Control subjects had to be free of a depressive disorder, apart from that inclusion and exclusion criteria were analogous to the patient sample.

### Procedures

Assessments took place 1) before the beginning of antidepressant treatment (baseline = BL), 2) 14 ± 1 days following onset of antidepressant treatment (T1) and 3) after additional 14 ± 1 days (T2, i.e. after 28 ± 1 days of medication). On each time point a German version of a structured interview^49^ was performed as basis for the assessment of depression severity in the HAMD-17. All interviews were performed blind to EEG-analyses. In order to obtain highest reliability in symptom scoring, a rater training for the interviewers (MD, CN) was performed by a clinically experienced physician (FMS). In those participants giving specific permission for a video recording of the interview (n = 58), the interviews were re-evaluated and evaluations of a clinically experienced physician (TS) blind to both time point of interview and subject interviewed were included into statistical analyses. The inter-rater (ICC = 0.983) and intra-rater reliabilities (ICC = 0.955) were calculated for 10 randomly selected interviews, showing good concordance. Therefore, in interviews of patients not agreeing to be videotaped, all interviews were performed and scored by the same interviewer. ‘Response’ was defined as reductions in HAMD-17-scores from BL to T2 ≥50%. Additionally, both depressed participants and healthy controls answered the BDI-II^50^.

According to the naturalistic design of the study, treatment was conducted to the therapists’ decision based on a therapeutic algorithm applied at the study centre^51^. In short, antidepressant therapy within the first 4 weeks of treatment regularly consisted of a monotherapy with either escitalopram or mirtazapine (N = 53). In those patients with a history of non-response to these two antidepressant agents (N = 5) treatment was conducted with an alternative antidepressant. In N = 7 cases, treatment with escitalopram or mirtazapine was combined with or substituted by another antidepressant listed in Table 1.

### EEG recordings

Within the patient sample, EEG recordings were performed at BL and T1. However, not all patients were available for or willing to participate in the T1 EEG-recording. Thus, BL-datasets of 65 patients (and of 65 healthy controls) and T1-datasets of 57 patients were included into statistical analyses. Fifteen minutes of resting-EEG with eyes closed were recorded between 8:00 a.m. and 2:00 p.m. Within patients, time of recording was not allowed to vary more than ± 1 h between BL and T1. During the EEG recording, participants were instructed to relax and not to fight a possibly occurring urge to fall asleep. The EEG was recorded with a 40 channel QuickAmp amplifier (Brain Products GmbH, Gilching, Germany) from 31 electrode sites according to an extended version of the international 10–20 system at a sampling rate of 1 kHz, referenced against common average using a low-pass filter at 280 Hz. Impedances were kept below 10 kΩ. Electrooculogram (EOG) electrodes were placed above the upper left eye and under the lower right eye.

### Assessment and classification of EEG-vigilance

EEG raw data were processed using BrainVision Analyzer 2.0 (Brain Products GmbH, Gilching, Germany). EEG raw data was filtered at 70 Hz (low-pass), 0.5 Hz (high-pass) and 50 Hz (notch-filter, range ± 2 Hz). EOG channels were visually screened for periods of open eyes which were excluded from further analysis. Eye movement artefacts were removed with an independent component analysis (ICA) approach by extracting 1–3 independent components that clearly represented vertical and horizontal eye movements. Likewise, persistent muscle artefacts were removed in the ICA approach. Afterwards, segments with remaining muscle, movement, eye and sweating artefacts were marked for exclusion from further analysis.

Using the freely available Vigilance Algorithm Leipzig 2.1 (VIGALL; http://www.uni-leipzig.de/~vigall/), each of the consecutive 900 one-second segments was attributed to one of seven different EEG-vigilance stages (for details refer to VIGALL 2.1 manual^52^):

- Stage 0 (corresponding to an activated state): defined by low amplitude, desynchronized, non-alpha activity in the absence of slow horizontal eye movements (SEMs);
- Stage A (corresponding to relaxed wakefulness) defined by dominant alpha activity and (further divided into sub-stages A1, A2, A3 according to the degree of alpha anteriorisation from occipital to more anterior cortices);
- Stage B (corresponding to drowsiness) with low amplitude non-alpha in the presence of SEMs (sub-stage B1) or prominent theta/delta activity (sub-stage B2/3);
- Stage C (characterising sleep onset) in case of occurrence of sleep spindles or K-complexes (all EEGs were visually screened and the respective EEG segments containing such graphoelements were marked manually before VIGALL application).

Next, VIGALL results were imported into a Microsoft Excel template with Visual Basic for Applications (VBA) macros which was used for a plausibility-check of stage 0-classification as recommended in the VIGALL manual. To avoid over-classification of 0-stages segments, stages 0 are to be reclassified as stage B1 if they occur in close proximity of B2/3- or C-stages even in the absence of SEMs, as SEMs are characteristic but not imperative for drowsiness patterns. Afterwards, the absolute amount and percentage (amount * 100/total number of non-artefact segments) of EEG-vigilance stages (stage 0, A, B and C, sub-stages A1, A2, A3, B1, B2/3) was calculated over the whole recording period and within blocks of 1-min duration.

### Statistics

To investigate differences of EEG-vigilance between groups we performed repeated measures ANOVAs with “outcome” (responders vs. non-responders) or “group” (depressed patients vs healthy controls) as between subject factors, “stage” (0, A1, A2, A3, B1, B2/3, C) and “recording time” (minutes 1–15 with 15 blocks of 1  min each) as within subject factor. To investigate differences of EEG-vigilance from BL to T1 between responders and non-responders repeated measures ANOVAs with “outcome” (responders vs. non-responders) as between subject factors, “stage” (0, A1, A2, A3, B1, B2/3, C) and “assessment” (BL vs. T1) as within subject factor were performed. Differences between groups concerning socio-demography, severities, history of disease and treatment were analysed using parametric tests (t tests) or non-parametric tests (e.g., chi-square tests, Mann-Whitney U tests) according to data level.

Effect sizes for group differences regarding EEG parameters were calculated using Cohen’s d^53^. The parameters with the highest effect sizes in the analyses between responders and non-responders were selected for calculating the prediction of response. To assess their accuracy rates, receiver operating characteristic (ROC) analyses were performed and corresponding area under the curve values (AUC) were computed. The sensitivity and specificity of the selected parameters were computed for different cut-off values. The Youden index was applied to select optimal cut-off scores^54^.

The IBM Statistical Package for the Social Sciences (SPSS) program version 20.0 for Windows was used for all statistical analyses. The significance level was set at p < 0.05.

## Additional Information

**How to cite this article:** Schmidt, F. M. *et al*. Brain arousal regulation as response predictor for antidepressant therapy in major depression. *Sci. Rep.* **7**, 45187; doi: 10.1038/srep45187 (2017).

**Publisher's note:** Springer Nature remains neutral with regard to jurisdictional claims in published maps and institutional affiliations.

## Supplementary Material

Supplementary Dataset 1

## Figures and Tables

**Table 1:** Sample characteristics (sociodemographic, clinical and EEG-related variables) between depressed patients and healthy controls (left) as well as responders and non-responders (right).

	Healthy controls	Depressed patients	*p value*	Responders	Non-Responders (N = 36)	*p value*
	(N = 65)	(N = 65)		(N = 29)		
**Sociodemographic variables**						
Age [years] (mean ± SD)	36.55 ± 12.21	36.28 ± 12.13	*0.897*^*a*^	35.79 ± 13.42	36.67 ± 11.43	*0.780*^*a*^
Sex (M/F)	32/33	32/33	*1.000*^*b*^	13/16	19/17	*0.524*^*b*^
Smoker [yes/no]	11/54	30/35	<*0.001*^*b*^	17/12	13/23	*0.070*^*b*^
**Current episode**						
Melancholic subtype [yes/no]	—	41/24	*NA*	17/12	24/12	*0.607*^*b*^
Atypical subtype [yes/no]	—	6/59	*NA*	2/27	4/32	*0.684*^*b*^
**History of disease**						
First/recurrent episode	—	34/31	*NA*	16/13	18/18	*0.804*^*b*^
Duration of disease [years] (mean ± SD)	—	3.83 ± 6.59	*NA*	3.22 ± 5.28	4.31 ± 7.64	*0.536*^*b*^
**Severities**						
HAMD-17 T1 [score] (mean ± SD)	—	22.09 ± 6.25	*NA*	21.10 ± 6.24	23.03 ± 5.92	*0.186*^*c*^
HAMD-17 T2 [score] (mean ± SD)	—	15.66 ± 7.69	*NA*	11.62 ± 7.93	19.26 ± 5.49	<*0.001*^*c*^
HAMD-17 T3 [score] (mean ± SD)	—	12.43 ± 6.85	*NA*	6.34 ± 3.32	17.59 ± 4.56	<*0.001*^*c*^
BDI-II T1 [score] (mean ± SD)	5.80 ± 6.05	29.28 ± 11.30	<*0.001*^*c*^	27.24 ± 12.26	30.97 ± 10.30	*0.175*^*c*^
BDI-II T2 [score] (mean ± SD)	—	24.43 ± 14.07	*NA*	18.90 ± 12.57	29.31 ± 13.36	*0.005*^*c*^
BDI-II T3 [score] (mean ± SD)	—	19.62 ± 12.05	*NA*	11.78 ± 8.78	27.17 ± 10.58	<*0.001*^*c*^
**Treatment**						
In-ward/out-patient clinic	—	44/13	*NA*	18/7	26/6	*0.787*^*b*^
In-ward and out-patient clinic	—	8	*NA*	4	4	*1.000*^*b*^
**Medication**						
Escitalopram	—	40	*NA*	18	22	*0.339*^*b*^
Mirtazapine	—	13	*NA*	8	5	*0.218*^*b*^
Others	—	12	*NA*	3	9	*0, 2*
Escitalopram + Mirtazapine	—	1	*NA*	1	0	*NA*
Escitalopram, Bupropione	—	2	*NA*	1	1	*NA*
Escitalopram + Quetiapine	—	1	*NA*	0	1	*NA*
Escitalopram + Olanzapine	—	1	*NA*	0	1	*NA*
Mirtazapine, Agomelatine	—	1	*NA*	0	1	*NA*
Mirtazapine, Sertraline	—	1	*NA*	0	1	*NA*
Sertraline	—	2	*NA*	1	1	*NA*
Duloxetine	—	2	*NA*	0	2	*NA*
Agomelatine	—	1	*NA*	0	1	*NA*
**EEG-recording conditions**						
Time of EEG recording [hh:min] (mean ± SD)	12:10 ± 2:49	11:22 ± 1:53	*0.063*^*a*^	11:15 ± 1:57	11:28 ± 1:51	*0.656*^*a*^
Coffee consumption prior to EEG [yes/no]	6/59	33/32	*0.020*^*b*^	17/12	16/19	*0.524*^*b*^
Time of coffee consumption [hh:ss] (mean ± SD)	10:00 ± 2:57	7:42 ± 1:43	*0.116*^*a*^	7:33 ± 2:15	7:51 ± 0:58	*0.626*^*a*^
Nicotine consumption prior to EEG [yes/no]	6/59	25/40	*0.007*^*b*^	15/14	11/25	*0.083*^*b*^
Time of nicotine consumption [hh:ss] (mean ± SD)	11:39 ± 1:10	10:20 ± 2:27	*0.093*^*a*^	9:41 ± 2:19	11:20 ± 2:26	*0.108*^*a*^
Proportion of artifacts [%] (mean ± SD)	0.80 ± 1.48	1.09 ± 3.04	*0.487*^*a*^	2.19 ± 2.26	1.18 ± 1.33	*0.026*^*a*^

Annotations: a = t-test; b = chi^2^ –test; c = Mann-Whitney-U-test.

**Table 2:** Comparisons in baseline EEG-vigilance between responders (N = 29) and non-responders (N = 36) to antidepressant therapy.

	Responders	Non-Responders	Main effect “group”	Main effect “recording time”	Interaction “time x group”
	(mean ± SE)	(mean ± SE)			
**Stage 0 [%]**	9.71 ± 2.15	14.23 ± 2.67	*F*_1,63_ = 1.630, *p* = 0.206	*F*_7,451_ = 0.959, *p* = 0.462	*F*_7,451_ = 0.334, *p* = 0.941
**Stage A [%]**	**71.35 ± 4.31**	**51.58 ± 4.93**	***F***_**1,63**_** = 8.622,** ***p***** = 0.005**	***F***_**4,248**_** = 18.781,** ***p***** < 0.001**	*F*_4,248_ = 0.853, *p* = 0.492
**Sub-stage A1 [%]**	**59.67 ± 4.8**	**44.67 ± 4.99**	***F***_**1,63**_** = 4.613,** ***p***** = 0.036**	***F***_**4,256**_** = 20.789,** ***p***** < 0.001**	*F*_4,256_ = 0.502, *p* = 0.737
**Sub-stage A2 [%]**	10.82 ± 2.81	5.69 ± 1.79	*F*_1,63_ = 2.54, *p* = 0.116	*F*_5,314_ = 1.116, *p* = 0.352	*F*_5,314_ = 1.060, *p* = 0.383
**Sub-stage A3 [%]**	**0.86 ± 0.33**	**1.22 ± 0.34**	*F*_1,63_ = 0.002, *p* = 0.963	***F***_**5,339**_** = 3.955,** ***p***** = 0.001**	*F*_5,339_ = 1.125, *p* = 0.347
**Stage B [%]**	**18.72 ± 5.11**	**32.64 ± 3.70**	***F***_**1,63**_** = 7.433,** ***p***** = 0.008**	***F***_**5,344**_** = 9.128,** ***p***** < 0.001**	*F*_5,344_ = 0.819, *p* = 0.546
**Sub-stage B1 [%]**	**11.64 ± 2.40**	**25.59 ± 3.44**	***F***_**1,63**_** = 10.033,** ***p***** = 0.002**	***F***_**6,393**_** = 5.083,** ***p***** < 0.001**	*F*_6,393_ = 1.317, *p* = 0.246
**Sub-stage B2/3 [%]**	7.07 ± 1.67	7.05 ± 1.71	*F1*_,63_ = 0.000, *p* = 0.987	***F***_**4,234**_** = 8.755,** ***p***** < 0.001**	*F*_4,234_ = 0.808, *p* = 0.513
**Stage C [%]**	0.22 ± 0.14	1.55 ± 0.74	*F*_1,63_ = 2.474, *p* = 0.121	*F*_3,182_ = 2.087, p = 0.106	*F*_3,182_ = 1.115, *p* = 0.343

**Table 3:** Changes in occurrence of EEG-vigilance stages from BL to T1 between responders (N = 27) and non-responders (N = 30) during antidepressant therapy.

	Responders	Non-Responders	Main effect “group”	Main effect “assessment”	Interaction “assessment x group”
	(mean ± SE)	(mean ± SE)			
**Stage 0 [%]**	**−4.129 ± 1.46**	**+6.47 ± 3.15**	***F***_**1,55**_** = 10.833,** ***p***** = 0.002**	*F*_1,55_ = 0.422, *p* = 0.518	***F***_**1,55**_** = 8.645,** ***p***** = 0.005**
**Stage A [%]**	−3.49 ± 4.87	+6.75 ± 4.23	***F***_**1,55**_** = 5.130,** ***p***** = 0.027**	*F*_1,55_ = 0.257, *p* = 0.614	*F*_1,55_ = 2.540, *p* = 0.117
**Sub-stage A1 [%]**	+1.89 ± 4.89	+5.41 ± 4.19	***F***_**1,55**_** = 4.018,** ***p***** = 0.049**	*F*_1,55_ = 1.295, *p* = 0.260	*F*_1,55_ = 0.302, *p* = 0.585
**Sub-stage A2 [%]**	**−5.40 ± 2.91**	**+1.32 ± 1.48**	*F*_1,55_ = 0.508, *p* = 0.479	*F*_1,55_ = 1.655, *p* = 0.204	***F***_**1,55**_** = 4.494,*****p***** = 0.039**
**Sub-stage A3 [%]**	+0.02 ± 0.23	+0.02 ± 0.30	*F*_1,55_ = 0.613, *p* = 0.437	*F*_1,55_ = 0.014, *p* = 0.907	*F*_1,55_ = 0.000, *p* = 0.995
**Stage B [%]**	**+7.86 ± 4.06**	**−12.06 ± 4.29**	*F*_1,55_ = 0.363, *p* = 0.549	*F*_1,55_ = 0.502, *p* = 0.482	***F***_**1,55**_** = 11.234,** ***p***** = 0.001**
**Sub-stage B1 [%]**	**+6.85 ± 3.50**	**−9.89 ± 3.24**	*F*_1,55_ = 1.037, *p* = 0.313	*F*_1,55_ = 0.409, *p* = 0.525	***F***_**1,55**_** = 12.353, p < 0.001**
**Sub-stage B2/3 [%]**	+1.01 ± 1.32	−2.17 ± 1.83	*F*_1,55_ = 0.189, *p* = 0.665	*F*_1,55_ = 0.255, *p* = 0.615	*F*_1,55_ = 1.910, *p* = 0.173
**Stage C [%]**	−0.24 ± 0.15	−1.15 ± 0.59	***F***_**1,55**_** = 2.074,** ***p***** = 0.155**	*F*_1,55_ = 4.803, *p* = 0.033	*F*_1,55_ = 2.074, *p* = 0.155
