# Supplementary material for: Brain arousal regulation as response predictor for antidepressant therapy in major depression
Source: Sci Rep. 2017 Mar 27;7:45187. doi: 10.1038/srep45187 (PMC5366924; doi:10.1038/srep45187)
Supplement: Supplementary Dataset 1 [file srep45187-s1.doc]

**Supplement to:**

‘Brain arousal regulation as response predictor for antidepressant therapy in major depression’

Authors: Frank M. Schmidt, Christian Sander, Marie-Elisa Dietz, Claudia Nowak, Thomas Schröder, Roland Mergl, Peter Schönknecht, Hubertus Himmerich, Ulrich Hegerl

**Comparisons of VIGALL 2.0 and VIGALL 2.1 on differences between patients with major depression and healthy subjects**

1. Analyses of EEG-vigilance between 30 un-medicated depressed patients and 30 non-depressed controls.

Original results on **VIGALL 1.0** are to be found in:

Hegerl, U., Wilk, K., Olbrich, S., Schoenknecht, P., Sander, C. Hyperstable regulation of vigilance in patients with major depressive disorder. *World J. Biol. Psychiatry.* 2012; 13: 436-446. doi: 10.3109/15622975.2011.579164.

The original analyses were performed with the VIGALL 1.0. In order to compare the two recent versions of the VIGALL, all analyses between the groups were re-run with the VIGALL 2.0 and VIGALL 2.1.

Conclusions:

1. The lower border for the VIGALL detection range was adapted from 2Hz to 3Hz in the VIGALL 2.1. Following that, the occurrence of sub-stage B2/3 was reduced throughout the 15-min measure compared to VIGALL 2.0. Importantly, the proportion of sub-stage B2/3 within the first minutes when only little of such low vigilance stage ought to be expected was markedly reduced from approx. 8 % in VIGALL 2.0. to 2 % in VIGALL 2.1, which more properly displays the physiologically later occurrence of sub-stage B2/3:

VIGALL 2.1

VIGALL 2.0


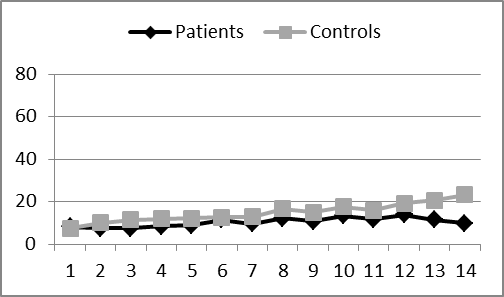

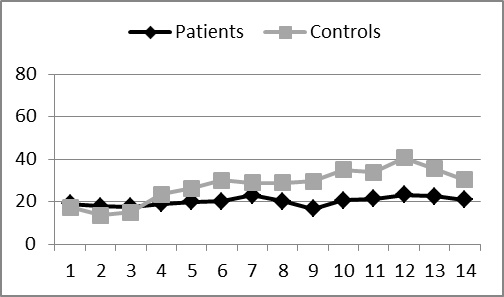


1. The modifications in the settings for the relative power threshold for EEG-vigilance sub-stage A1 in the VIGALL 2.1 resulted in more prominent differences in the regulation between the two groups. This is in good accordance with the concept of an upregulation of brain arousal within major depression: Depressed patients and healthy controls show an occurrence of ~65 % within the first minutes. Whereas the healthy controls show a physiological decline of sub-stage A1 over time down to 30%, the proportion of sub-stage A1 remains stable within the depressed subjects with 56% in minute 15. Further, the more prominent proportion of sub-stage A1 (compared to a higher proportion of sub-stages A2 and A3 in VIGALL 2.0) at the beginning of the recording period better reflects the preponderance of occipital alpha physiologically observed during rest:

|  | **VIGALL 2.1** |  |  | **VIGALL 2.0** |  |  |
| --- | --- | --- | --- | --- | --- | --- |
| **Sub-stage A1** | **Test statistics** | **p values** | **partial eta^2^** | **Test statistics** | **p values** | **partial eta^2^** |
|  |  |  |  |  |  |  |
| **Repeated measures** |  |  |  |  |  |  |
| **ANOVA** |
| **Time** | **F= 22.054** | **<0.001** | 0.275 | **F= 15.313** | **<0.001** | 0.209 |
| **Group** | **F = 5.236** | **0.026** | 0.083 | **F = 7.481** | **0.008** | 0.114 |
| **Time x Group** | **F = 6.458** | **<0.00001** | 0.10 | **F = 3.696** | **0.005** | 0.06 |

VIGALL 2.1

VIGALL 2.0


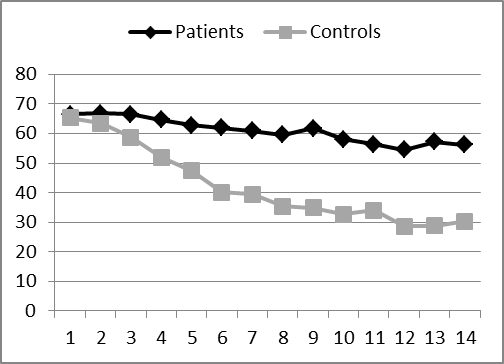

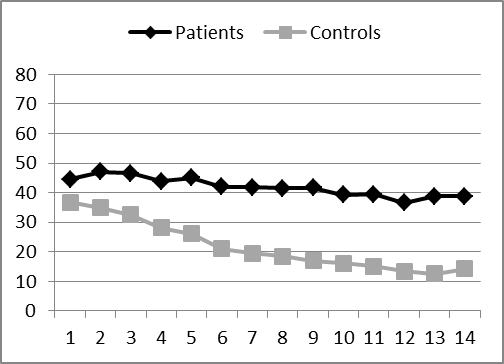


1. Following the plausibility check on the separation of stage W and sub-stage B1, VIGALL 2.1 showed a lower proportion of stage W and a higher proportion of sub-stage B1 than VIGALL 2.0. The latter was found significant for a ‘time x group’ interaction for sub-stage B1 with the VIGALL 2.1 only:

|  | **VIGALL 2.1** |  |  | **VIGALL 2.0** |  |  |
| --- | --- | --- | --- | --- | --- | --- |
| **Sub-stage B1** | **Test statistics** | **p values** | **partial eta^2^** | **Test statistics** | **p values** | **partial eta^2^** |
|  |  |  |  |  |  |  |
| **Repeated measures** |  |  |  |  |  |  |
| **ANOVA** |
| **Time** | **F= 5.401** | **<0.001** | 0.085 | **F= 2.268** | **0.006** | 0.038 |
| **Group** | **F = 1.745** | **0.192** | 0.029 | **F = 1.275** | **0.263** | 0.022 |
| **Time x Group** | **F = 2.623** | **0.028** | 0.043 | **F = 1.559** | **0.180** | 0.026 |

VIGALL 2.1

VIGALL 2.0


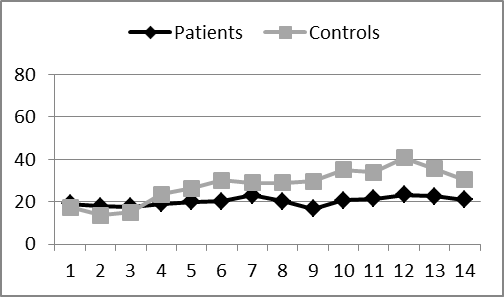

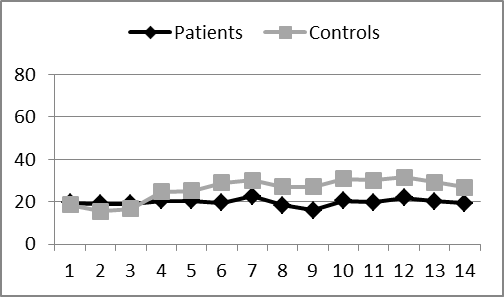


1. Concerning the mean vigilance, VIGALL 2.1 analyses showed better results than the VIGALL 2.0 analyses on ‘group’ effect (depressed vs. controls) as well as ‘time x group’ interactions in p values and partial eta^2^ values:

|  | **VIGALL 2.1** |  |  | **VIGALL 2.0** |  |  |
| --- | --- | --- | --- | --- | --- | --- |
| **Mean vigilance** | **Test statistics** | **p values** | **partial eta^2^** | **Test statistics** | **p values** | **partial eta^2^** |
|  |  |  |  |  |  |  |
| **Repeated measures  ANOVA** |  |  |  |  |  |  |
| **Time** | **F= 13.785** | **<0.001** | 0.192 | **F= 9.998** | **<0.001** | 0.147 |
| **Group** | **F = 5.776** | **0.019** | 0.091 | **F = 5.277** | **0.025** | 0.083 |
| **Time x Group** | **F = 4.341** | **0.001** | 0.070 | **F = 3.779** | **0.005** | 0.061 |


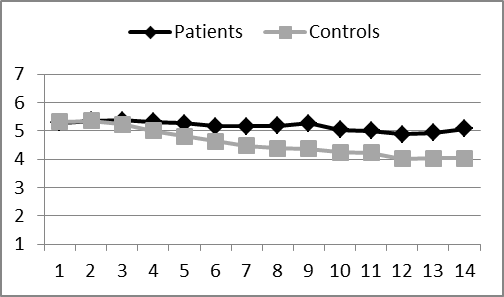

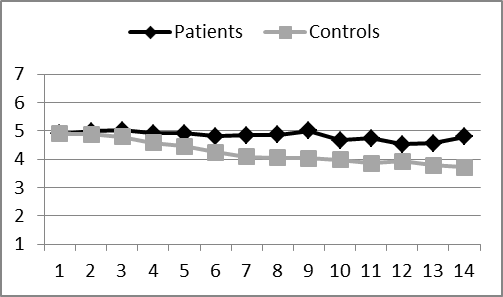


VIGALL 2.1

VIGALL 2.0

1. Analyses of EEG-vigilance between 60 un-medicated depressed patients and 129 non-depressed controls.

Original results on differences between groups on **VIGALL 2.0** analyses are to be found in:

^1^ Schmidt FM, Pschiebl A, Sander C, Kirkby KC, Thormann J, Minkwitz J, Chittka T, Weschenfelder J, Holdt LM, Teupser D, Hegerl U, Himmerich H. Impact of Serum Cytokine Levels on EEG-Measured Arousal Regulation in Patients with Major Depressive Disorder and Healthy Controls. Neuropsychobiology. 2016; 73: 1-9. doi: 10.1159/000441190

Conclusions:

Compared with the statistics conducted on VIGALL 2.0 analyses, the VIGALL 2.1 show improved F and p-values for the **differences in the overall occurrence** of stage A and stage B between the two groups. Simultaneously, partial eta^2^ for effect sizes improved for both stages (upper rows). The proportion of sub-stage A1 increased, whereas the proportion of sub-stage B2/3 decreased. Analyses on the **mean vigilance** (lower rows) showed a more significant ‘group’ effect (depressed vs. controls) as well as ‘time x group’ interactions with VIGALL 2.1 than with VIGALL 2.0, both with higher partial eta^2^ values.

Revised calculations on **VIGALL 2.1** analyses (analyses and table adapted from ^1^):

|  | **Depressed** | **Non-depressed** | **Test statistics** | **p values** | **partial eta^2^** |
| --- | --- | --- | --- | --- | --- |
|  | **value (± SD)** | **value (± SD)** |  |  |  |
|  |  |  |  |  |  |
| **Stage W [%]** | 8.78 (± 13.23) | 8.63 (± 12.14) | F=0.006 | 0.939 | 0.000 |
| **Stage A [%]** | 59.31 (± 29.06) | 47.55 (± 28.80) | **F=6.771** | **0.010** | 0.035 |
| **Substage A1 [%]** | 46.50 (± 27.90) | 38.60 (± 26.39) | F=3.530 | 0.062 | 0.019 |
| **Substage A2 [%]** | 11.79 (± 16.85) | 7.23 (± 12.17) | **F=4.435** | **0.037** | 0.023 |
| **Substage A3 [%]** | 1.01 (± 1.61) | 1.71 (± 3.03) | F=2.811 | 0.095 | 0.015 |
| **Stage B [%]** | 31.48 (± 27.14) | 32.02 (± 24.27) | **F=6.865** | **0.010** | 0.036 |
| **Substage B1 [%]** | 24.79 (± 23.43) | 32.02 (± 27.14) | F=3.706 | 0.056 | 0.020 |
| **Substage B2/3 [%]** | **6.70 (± 9.59)** | **10.69 (± 14.07)** | **F=3.975** | **0.048** | 0.021 |
| **Stage C [%]** | 0.42 (± 1.47) | 1.10 (± 3.77) | F=1.800 | 0.181 | 0.010 |
| **Repeated measures ANOVA** |  |  |  |  |  |
| **Time** |  |  | **F= 56.212** | **<0.001** | 0.232 |
| **Group** |  |  | **F = 5.419** | **0.021** | 0.028 |
| **Time x Group** |  |  | **F = 4.164** | **0.011** | 0.022 |
| **Post-hoc minutes 1-3** | 5.34 (± 0.87) | 5.27 (± 0.87) | F = 0.247 | 0.620 | 0.001 |
| **Post-hoc minutes 4-6** | 5.13 (± 0.89) | 4.88 (± 1.09) | F = 2.352 | 0.127 | 0.012 |
| **Post-hoc minutes 7-9** | 4.98 (± 0.93) | 4.56 (± 1.15) | **F = 6.243** | **0.013** | 0.032 |
| **Post-hoc minutes 10-12** | 4.85 (± 1.01) | 4.35 (± 1.08) | **F = 8.979** | **0.003** | 0.046 |
| **Post-hoc minutes 13-15** | 4.74 (± 1.12) | 4.73 (± 1.01) | **F = 5.955** | **0.016** | 0.031 |
